# Autoinactivation of the Stargazin–AMPA Receptor Complex: Subunit-Dependency and Independence from Physical Dissociation

**DOI:** 10.1371/journal.pone.0049282

**Published:** 2012-11-14

**Authors:** Artur Semenov, Tommi Möykkynen, Sarah K. Coleman, Esa R. Korpi, Kari Keinänen

**Affiliations:** 1 Department of Biosciences, Division of Biochemistry and Biotechnology, University of Helsinki, Helsinki, Finland; 2 Institute of Biomedicine, Pharmacology, University of Helsinki, Helsinki, Finland; Virginia Commonwealth University, United States of America

## Abstract

Agonist responses and channel kinetics of native α-amino-3-hydroxy-5-methyl-4-isoxazole propionic acid (AMPA) receptors are modulated by transmembrane accessory proteins. Stargazin, the prototypical accessory protein, decreases desensitization and increases agonist potency at AMPA receptors. Furthermore, in the presence of stargazin, the steady-state responses of AMPA receptors show a gradual decline at higher glutamate concentrations. This “autoinactivation” has been assigned to physical dissociation of the stargazin-AMPA receptor complex and suggested to serve as a protective mechanism against overactivation. Here, we analyzed autoinactivation of GluA1–A4 AMPA receptors (all flip isoform) expressed in the presence of stargazin. Homomeric GluA1, GluA3, and GluA4 channels showed pronounced autoinactivation indicated by the bell-shaped steady-state dose response curves for glutamate. In contrast, homomeric GluA2i channels did not show significant autoinactivation. The resistance of GluA2 to autoinactivation showed striking dependence on the splice form as GluA2-flop receptors displayed clear autoinactivation. Interestingly, the resistance of GluA2-flip containing receptors to autoinactivation was transferred onto heteromeric receptors in a dominant fashion. To examine the relationship of autoinactivation to physical separation of stargazin from the AMPA receptor, we analyzed a GluA4-stargazin fusion protein. Notably, the covalently linked complex and separately expressed proteins expressed a similar level of autoinactivation. We conclude that autoinactivation is a subunit and splice form dependent property of AMPA receptor-stargazin complexes, which involves structural rearrangements within the complex rather than any physical dissociation.

## Introduction

Cellular localization and functional properties of α-amino-3-hydroxy-5-methyl-4-isoxazolepropionic acid (AMPA) receptors are strongly influenced by transmembrane AMPA receptor regulatory proteins (TARP) (for reviews, see [1], [2], [3]). To date, six homologous TARP, named as γ2–5, γ7, andγ8, have been identified and found to participate in the regulation of neuronal and glial AMPA receptors [4], [5], [6], [7], [8]. Stargazin (γ2), the founding and best characterized member of TARP family, enhances AMPA receptor function by at least two distinct mechanisms. It is a key operator in AMPA receptor trafficking by promoting receptor transport to cell surface and stabilization to synaptic membrane [4], [5], [9]. Stargazin also enhances the ligand-gated channel function of AMPA receptors by increasing agonist affinity, decreasing densensitization, and by weakening polyamine block of Ca^2+^-permeable AMPA receptors at depolarized potentials [10], [11], [12], [13], [14]. Moreover, association with stargazin leads to profound changes in agonist and antagonist pharmacology of AMPA receptors [15], [16], [17], [18]. Due to this profound modulation and the near-stoichiometric association of native AMPA receptors with stargazin and related TARP [19], the complex between TARP and AMPA receptor has become a critical subject for studies addressing the structure and function of AMPA receptors.

An interesting new facet of TARP modulation was revealed by the recent demonstration that in the presence of stargazin, steady-state glutamate responses of AMPA receptors exhibit an aberrant decline at concentrations ≥ 100 µM [20]. This phenomenon, termed autoinactivation, was linked to a time- and concentration -dependent uncoupling of stargazin -receptor interaction, via dissociation of the complex [20]. In the present study, we have investigated stargazin-dependent autoinactivation in GluA1-4 AMPA receptors. We demonstrate the presence of striking subunit- and splice variant-dependent differences in autoinactivation and present data to support the notion that autoinactivation and physical dissociation of stargazin-AMPA receptor complex are separate processes.

## Results

### Subunit-dependent differences in autoinactivation

**Figure 1:**
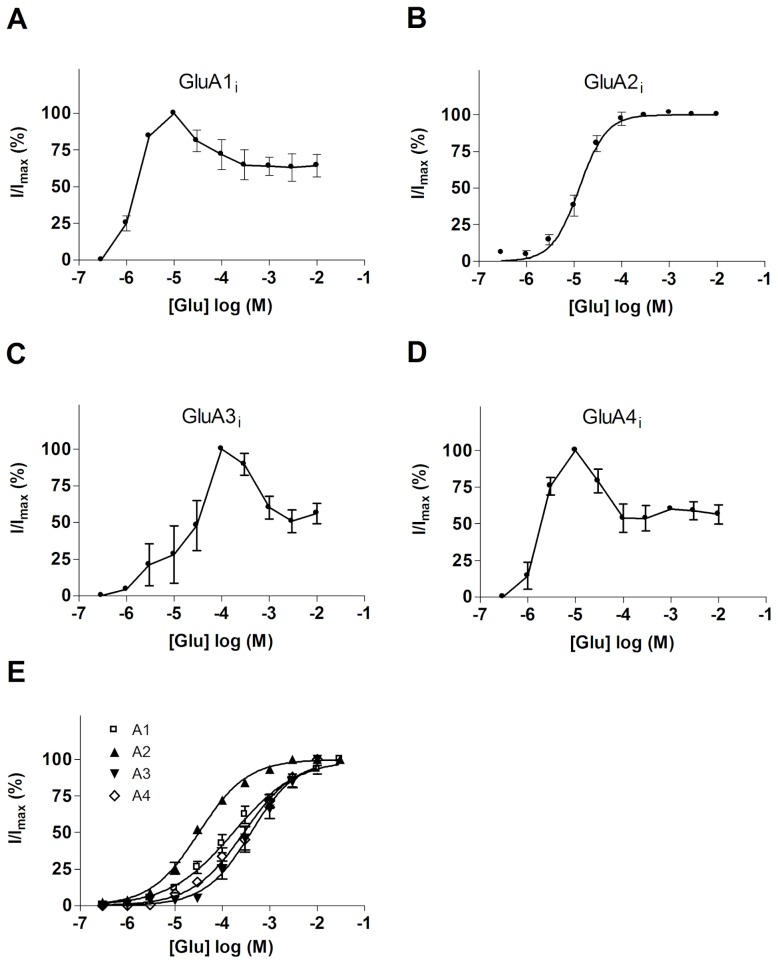
Subunit-dependent differences in stargazin-dependent autoinactivation of AMPA receptors. (***A–D***) Steady-state concentration-response curves of l-glutamate-triggered current responses of homomeric GluA1i (*A*), GluA2i (*B*), GluA3i (*C*) GluA4i (*D*) channels coexpressed with stargazin. (***E***) Concentration-response curves of l-glutamate-triggered peak responses of homomeric GluA1-4i receptors. Currents were normalized to the maximal response obtained for each channel. The points represent the mean ± S.E.M of recordings from 5–6 cells from a typical experiment.

AMPA receptor subunits GluA1-4 were expressed together with stargazin in transiently transfected HEK293 cells, and the resulting homomeric channels were characterized by using whole cell patch clamp recordings. All subunits were of the flip isoform (GluA1-4i), and the GluA2 subunit was edited (R) at the Q/R site. With GluA1i, GluA3i, and GluA4i, a bell-shaped relation was observed between the steady-state current amplitudes and glutamate concentration, indicative of the presence of autoinactivation (Figure 1A,C,D). Glutamate concentration yielding the highest steady-state current differed slightly between subunits: for GluA1i and GluA4i, the maximal steady-state current response was obtained at 10 µM, whereas GluA3i channels gave the highest response at 100 µM glutamate concentration. For all three, steady-state responses obtained at millimolar range (1–10 mM glutamate) corresponded to 50–60% of the highest steady-state response obtained at micromolar concentrations. In striking contrast to the three other homomeric channels, GluA2i consistently produced ordinary sigmoid concentration-response curves with no sign of decline in steady-state current amplitudes at the millimolar range (Figure 1B). Unlike the variation in the dose-response relationships of steady-state currents, the peak current responses of all four homomeric receptors gradually increased with increasing glutamate concentration (Figure 1E), fully consistent with the suggestion that autoinactivation represents time-dependent uncoupling of stargazin-modulation from AMPA receptors [20]. These findings indicate that autoinactivation of AMPA receptors is a subunit- and splice form-dependent property: homomeric GluA1i, GluA3i, and GluA4i channels exhibit robust autoinactivation, while GluA2i under similar conditions shows no significant autoinactivation.

### Isoform-dependent autoinactivation in GluA2 channels

**Figure 2:**
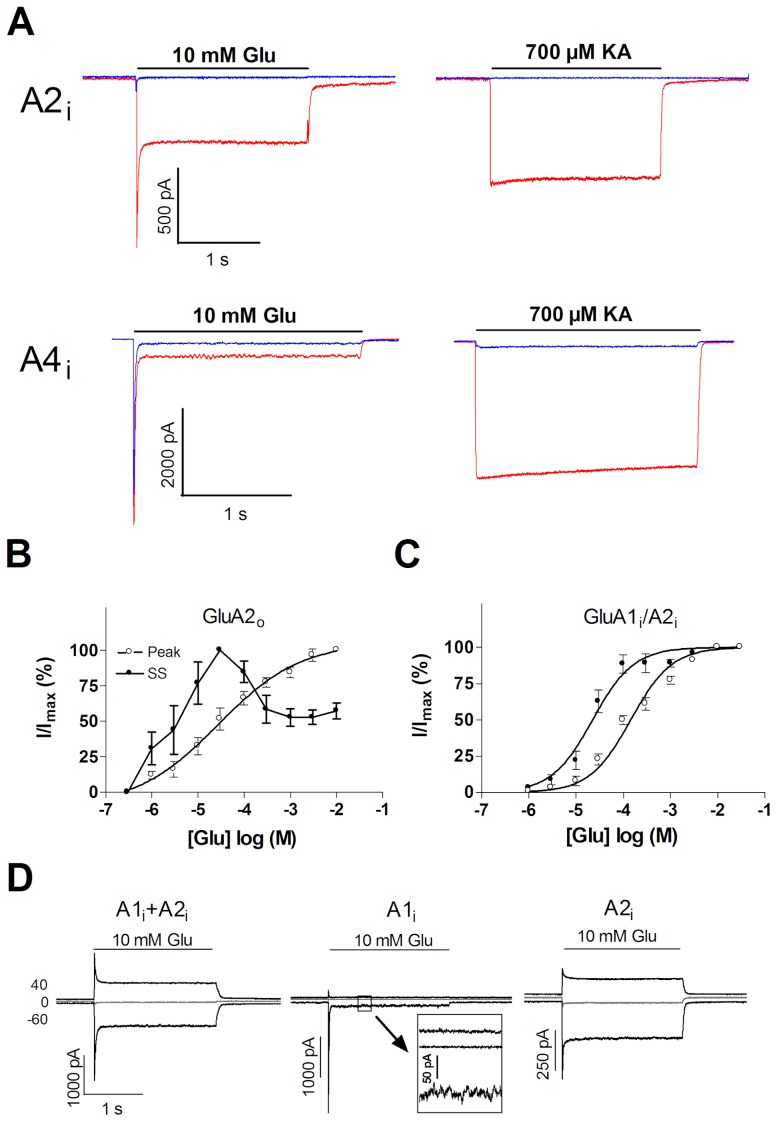
Absence of autoinactivation in homomeric and heteromeric GluA2 channels. (***A***) Representative current traces from patch clamp recordings of homomeric GluA2i and GluA4i channel responses to glutamate (10 mM) and kainate (0.7 mM) in the presence (red traces) and in the absence (blue traces) of stargazin. (***B***) Concentration-response curves of peak (open circles) and steady state (filled circles) l-glutamate responses of homomeric GluA2o channels coexpressed with stargazin. (***C***) Concentration-response curves of peak (open circles) and steady state (filled circles) l-glutamate responses of heteromeric GluA1i/A2i channels coexpressed with stargazin; (***D***) Rectification properties of heteromeric GluA1i/GluA2i (left), homomeric GluA1i (middle), and homomeric GluA2i (right) channels, all in the presence of stargazin. Representative traces of glutamate-evoked currents at holding potentials of +40 mV, 0 mV, and −60 mV are shown. The inset shows an enlarged view of GluA1i steady-state currents.

The striking absence of autoinactivation in GluA2i prompted us to characterize GluA2 channels further. When expressed alone, without stargazin, GluA2i showed only minimal current responses to glutamate and kainate. However, in the presence of stargazin, GluA2i gave robust responses, comparable in amplitude to those produced by GluA4i (Figure 2A). These findings are in agreement with the poor channel activity of homomeric Q/R-edited GluA2 channels, and also exclude a lack of interaction with stargazin as a (trivial) explanation for the resistance of GluA2i to autoinactivation. A previous study suggested that GluA2o receptors exhibit autoinactivation [20], prompting us to determine whether the discrepant behavior of GluA2i is related to the flip/flop isoform. As shown in Figure 2B, GluA2o channels coexpressed with stargazin exhibit robust autoinactivation, contrary to GluA2i (cf. Figure 1B), indicating that the flip/flop exon is a key determinant of autoinactivation in GluA2 receptors.

GluA2 subunit is a component in the majority of native AMPA receptors. Thus, we tested whether the conspicuous absence of autoinactivation in homomeric GluA2i channels is also manifested in a heteromeric setting. For this purpose, glutamate responses were registered from cells coexpressing both GluA2i and GluA1i with stargazin. Steady-state responses recorded from these cells rose gradually with increasing agonist concentrations and saturated at millimolar range, suggesting that autoinactivation is either absent or strongly reduced in GluA1i/A2i channels (Figure 2C). EC_50_ values determined for glutamate peak responses of GluA1i/A2i and GluA1i were very similar and distinct from GluA2i; GluA1i/A2i: 143 µM (95% confidence interval: 121–171 µM), GluA1i: 165 µM (138–216 µM), GluA2i: 32.3 µM (28.2-36.6 µM) (Figure S1). However, glutamate-triggered current responses recorded at three holding potentials, −60 mV, 0 mV, and +40 mV, showed strong inward rectification in GluA1i channels, whereas the responses from GluA1i/A2i and homomeric GluA2i channels indicated a more linear current-voltage relation (Figure 2D). Rectification index, I(−60 mV)/I(+40 mV), was 16.0±5.8 (n = 5) for GluA1 alone, 1.41±0.17 (n = 8) for GluA2i alone, and 1.39±0.41 (n = 7) for GluAi/A2i receptors (P<0.001 for the difference between GluA1i and either GluA2i or GluA1i/A2i). The unique functional profile of GluA1i/A2i channels suggests that the potential contribution of any separate homomeric GluA1i or GluA2i channel populations to the current responses, and thus, to the apparent absence of autoinactivation observed in GluA1i/A2i -expressing cells is minor. Therefore, the presence of GluA2i subunit can confer substantially reduced sensitivity to stargazin-dependent autoinactivation of heteromeric AMPA receptors, at least in the case of GluA1i/A2i heteromers.

### Autoinactivation in AMPA receptor - stargazin fusion protein

**Figure 3:**
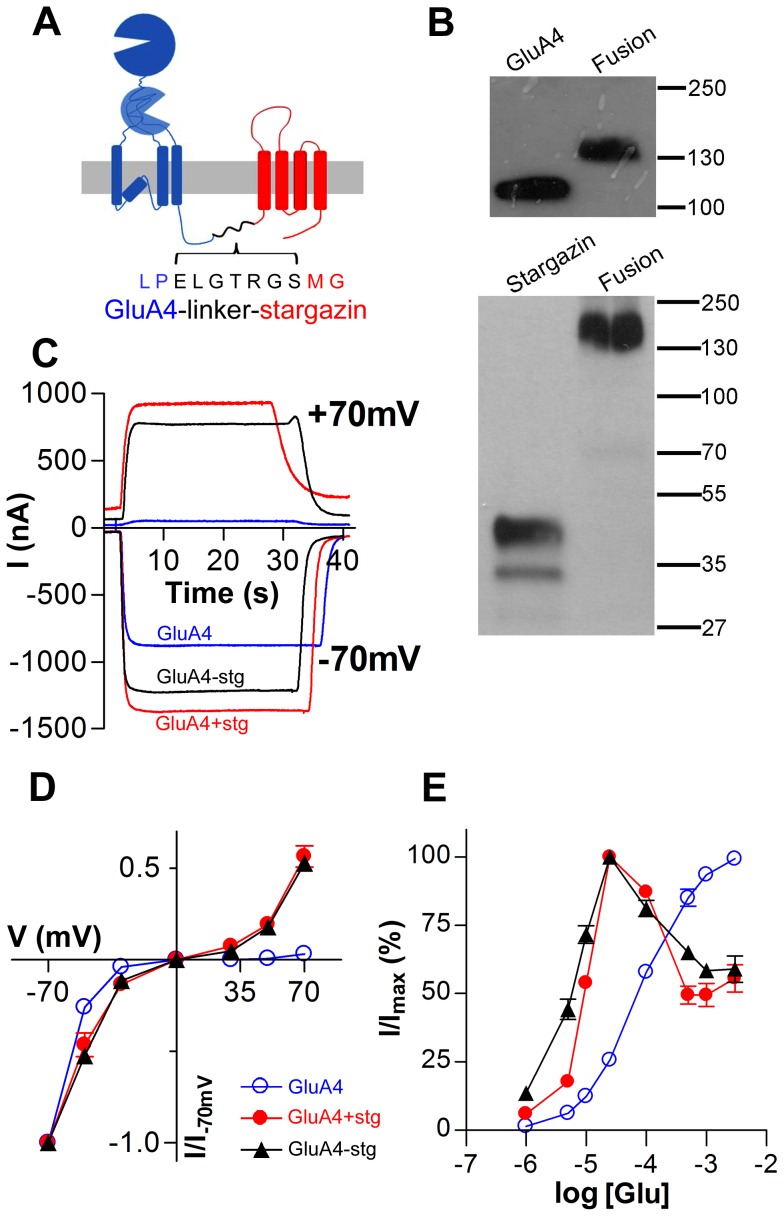
Autoinactivation of GluA4i - stargazin complex is not affected by covalent linkage between the proteins. (***A***) Schematic structure of GluA4i, stargazin and GluA4i-stargazin fusion protein. Amino acid sequence of the linkage between the carboxyterminus of GluA4 and the aminoterminus of stargazin is shown below the cartoon. (***B***) Western blot of GluA4i, stargazin and GluA4i-stargazin fusion protein detected by using anti-Flag (upper panel) and anti-stargazin (lower panel) antibodies. Molecular size markers (in kilodaltons) are indicated at the right. (***C–D***) Current-voltage relations of responses to l-glutamate (1 mM) in oocytes expressing the covalent GluA4i-stargazin fusion protein (black), and GluA4i expressed alone (blue) or in the presence of stargazin (red). (***E***) Concentration-response curves of l-glutamate -triggered currents in oocytes expressing the covalent GluA4i-stargazin fusion protein (black), and GluA4i expressed alone (blue) or in the presence of stargazin (red). The data represent the mean ± S.E.M of recordings from 3–9 oocytes.

We studied the dependence of autoinactivation on physical dissociation of AMPA receptor and stargazin by using a covalently bound fusion protein in which the C-terminus of GluA4i is linked to the N-terminus of stargazin by a short linker peptide (Figure 3A). Potentially, such a design would allow the formation of a functional complex between stargazin (stg) and the AMPA receptor and strongly drive the binding equilibrium towards the complex by maintaining high local concentrations of the components. Immunoblots detecting the N-terminal Flag epitope present in GluA4i and GluA4i-stg, and with an antibody specific for stargazin C-terminus, revealed protein products of expected size and the absence of any significant degradation products (Figure 3B). The electrophysiological properties of the fusion protein were preliminarily characterized by whole-cell patch clamp recordings, and then, in more detail and with similar results, by two-electrode voltage-clamp on cRNA-injected *Xenopus* oocytes, a convenient system for the analysis of steady-state responses.

First, we analyzed the I/V -relations of glutamate responses in order to ascertain the ability of the fusion protein to reproduce the basic functional properties of GluA4i coexpressed with stargazin. The inward rectification of GluA4i channels expressed in the absence of stargazin was significantly attenuated in the presence of coexpressed stargazin in the oocytes in agreement with earlier findings [14] (Figure 3C,D). Importantly, this attenuation of inward rectification was reproduced by covalently linked stargazin in an indistinguishable fashion from that observed with separately expressed proteins. Next, we measured the concentration-dependency of steady-state glutamate responses to reveal the presence or absence of autoinactivation. Current amplitudes recorded from oocytes expressing GluA4i alone showed a regular saturating concentration-dependency, whereas in the presence of stargazin, a significant decline of responses occurred as millimolar concentrations were approched, indicative of autoinactivation (Figure 3E). Again, the fusion protein and coexpressed proteins behaved in an indistinguishable manner, strongly suggesting that autoinactivation can occur in the absence of physical dissociation of stargazin -receptor complex. Based on the initial rising phase of dose-response curves, both the separately expressed and covalently linked stargazin caused a similar shift to the left, indicative of an increased glutamate potency consistent with earlier findings [10], [11], [12]. Finally, we examined by immunoprecipitation whether the noncovalent complex between GluA4i and stargazin is sensitive to glutamate-induced dissociation. GluA4i and stargazin, solubilized from transfected HEK293 cells, were immunoprecipitated by using an antibody against the N-terminal Flag tag, present in GluA4i [21] in the presence of 10 mM l-glutamate (a concentration which causes maximal autoinactivation; cf. Figure 1D), in the presence of 10 mM d-glutamate, and in the absence of glutamate. Stargazin immunoprecipitated with GluA4i to the same extent under all these conditions (Figure S2), indicating that the physical association of recombinant GluA4i with stargazin is not significantly affected by the presence of glutamate under the experimental conditions used.

## Discussion

Autoinactivation, the time-dependent uncoupling of stargazin-dependent augmentation of glutamate responses, was described and initially characterized in both native and recombinantly expressed AMPA receptors in a recent study [20]. Our study confirms the presence of robust autoinactivation in the stargazin-complexes of homomeric GluA1i, GluA3i and GluA4i channels, and also shows that, surprisingly, homomeric GluA2 channels exhibit very little autoinactivation under the same conditions. In further experiments, the flop isoform of GluA2 showed marked autoinactivation. Although we examined only the flip isoforms of non-GluA2 subunits, the earlier study suggested that autoinactivation is present in GluA1o and GluA2o, and may be weaker in GluA2i, but the latter finding was not characterized further [20]. However, they used data from two diagnostic glutamate concentration points, rather than a full dose-response curve as determined in the present study. Flip/flop-isoform-dependent differences in the modulation of the desensitization kinetics of homomeric AMPA receptors by stargazin have been previously reported: generally, the effects on flop-isoform receptors have been stronger than the effects on the corresponding flip receptors [15], [16], [22].

Earlier studies have shown that the cytoplasmic tail of stargazin is crucial for its trafficking role, whereas the extracellular loop between the first and second transmembrane segments is the major modulator of receptor function [12], [13], [23], [24]. Presumably, mutual interactions between these structures and the respectively located domains of AMPA receptors make critical contributions to the modulation. This view is supported by the importance of the extracellular ligand-binding domain of AMPA receptor for the stargazin modulation [25], and by the essential role of the flip/flop cassette (part of the ligand-binding domain) in determining the propensity of GluA2 to undergo stargazin-dependent autoinactivation as reported here. The unique absence of significant autoinactivation in GluA2i channels prompts future studies to identify which one(s) of the nine amino acid differences between the flip and flop variants account for the remarkable difference between the isoforms. Clearly, the absence of a comparable flip/flop-difference in GluA1, and possibly in other non-GluA2 subunits, implies that the flip-specific resistance to autoinactivation is manifested only in the unique structural context of the GluA2 receptor. Interestingly, recent analysis of GluA1/K2 chimeras showed that the cytosolic carboxylterminal tail of GluA1 is required for autoinactivation [20], implying that cytosolic elements and interactions make important contributions to stargazin-dependent channel modulation, a view supported by another recent study [26]. Alternatively, the arginine residue in the pore loop of the edited GluA2 subunit may be important; mutations at the Q/R site of GluA1 subunit have been reported to exert strong effects on stargazin-dependent modulation of desensitization [27]. At this stage, it can be concluded that the functional coupling between stargazin and AMPA receptor is critically dependent on both extracellular and intracellular interactions. High-resolution structural information on TARP-receptor complex is eagerly awaited in order to better resolve this issue.

It is important to note the high macroscopic currents mediated by homomeric Q/R-edited GluA2 channels when expressed in the presence of stargazin, an observation reported earlier [10], [13]. The existence of minor populations of native homomeric GluA2 receptors is commonly overlooked in the literature, presumably based on the weak channel activity of homomeric GluA2(R) receptors together with reported poor trafficking to cell surface of homomeric GluA2(R) channels [28]. However, high surface expression of GluA2(R) homomers has been observed in other studies (e.g. [27], [29], this study) and the presence of active synaptic GluA2 homomers has been demonstrated, at least under conditions where the expression of other subunits has been reduced [30]. Irrespective of the physiological relevance of GluA2 homomers, our finding that the GluA2i subunit may endow heteromeric GluA1i/A2i receptors with an apparent resistance to autoinactivation is important. As autoinactivation may act as a buffering mechanism against excitotoxicity [20], the present results suggest that GluA2i subunit content of AMPA receptors may be one of the factors determining the sensitivity of neurons to damage caused by prolonged exposure to glutamate.

Autoinactivation reflects the uncoupling of stargazin modulation from the receptor channel, but the underlying molecular mechanism is presently unclear. In principle, the phenomenon may be caused by physical dissociation of the receptor-stargazin complex or it may be caused by more subtle structural alterations which keep the complex intact but lead to loss of the modulatory effect. There is controversy regarding the stability of stargazin (TARP) - AMPA receptor complexes. Both the native and recombinantly expressed complex have been reported to be readily disrupted by exposure to glutamate [20], [23], but in other studies, rapid agonist-driven dissociation has not been observed ([31], this study). We found that a fusion protein which links the carboxylterminus of GluA4i to the N-terminus of stargazin shows strikingly similar autoinactivation to that observed in the case of separately expressed proteins. This finding, together with stability of the immunocomplex in the presence of glutamate, argues against a direct relation between physical dissociation and autoinactivation. In contrast to our results, covalent linkage between GluA1o and stargazin was reported to abolish autoinactivation [20]. The reason for this discrepancy is not clear, but differences in the design of the fusion protein remain a possibility. In particular, the shorter (two amino acid residues) linker used in GluA1o-stargazin fusion may enforce a more rigid structure to the complex than the seven-residue linker used in the present study. Alternatively, the conformational freedom may differ between GluA1o and GluA4i when covalently linked to stargazin.

**Figure 4:**
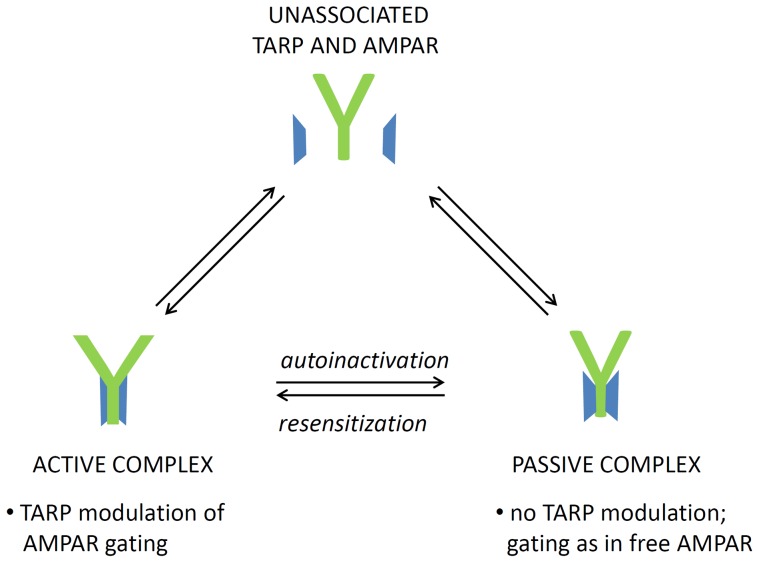
Active and passive AMPA receptor -TARP complexes. AMPA receptor (green Y) and TARP (blue trapezoid) are shown in schematic fashion. The arrows indicate the potential interconversions between the different states.

Based on research literature and our current results, we envision the stargazin-AMPA receptor complex to exist in (at least) two distinct states, designated here as active and passive states, depending on the presence of TARP modulation of channel gating (Figure 4). Autoinactivation represents the relaxation of the active TARP complex into the passive state in the presence of glutamate: in GluA2i-containing channels, the active state is remarkably stable thus inhibiting the autoinactivation process. This interpretation is consistent with the recent suggestion that autoinactivation is caused by functional rather than physical uncoupling [32]. Furthermore, resensitization occurring in AMPA receptor complexes with TARPs γ4, γ7, and γ8 [1], [6] although not with γ2/stargazin, would correspond to the reverse process. The active and passive states would be in equilibrium with the separate proteins, but - as judged from robust copurification of TARP with AMPA receptors from native and recombinant sources [19] - the equilibrium favours the complex. In addition to GluA2 splice form, the relative stability of the active and passive TARP complexes may depend on carboxyl-terminal interactions [20], may show differences between agonists, and can be regulated by interactions with additional regulatory proteins like cornichons [1], [33].

### Conclusion

Our results show that autoinactivation, the functional uncoupling of stargazin modulation of glutamate responses, is a subunit and splice form-dependent property of AMPA receptors: remarkably, homomeric GluA2i and GluA2i-containing heteromeric receptors show no or very little autoinactivation. Autoinactivation is not significantly influenced by covalent linkage between stargazin and AMPA receptor, suggesting that it is caused by relaxation of stargazin-AMPA receptor complex into a non-modulated state rather than by physical dissociation.

## Materials and Methods

### DNA constructs

Expression constructs encoding N-terminal Flag-tagged full-length rat AMPA receptor cDNAs in pcDNA3.1 (Stratagene) have been described [29], [34]. N-terminally Flag-tagged GluA4i-stargazin fusion construct was generated by overlap-extension PCR [35] using primers which introduced a linker sequence Glu-Leu-Gly-Thr-Arg-Gly-Ser between the carboxyl-terminal amino acid residue 902 (Pro) in GluA4 and the aminoterminal methionine in stargazin coding sequence. The fusion protein construct was cloned in pXOOM, a dual vector suitable for expression in mammalian cells and for generation of cRNA by *in vitro* transcription with T7 RNA polymerase [36]. GluA4i and stargazin coding sequences were also separately subcloned from pcDNA3.1 vector into pXOOM. All new constructs were verified by restriction mapping and by sequencing through the PCR amplified regions. The expression plasmid encoding human stargazin cDNA in pcDNA was a generous gift from John L. Black III (Mayo Medical School, Rochester, MN). Plasmid pEGFP-C1 (Clontech).

### Cell culture and transfection

HEK293 cells (Americal Type Culture Collection, CRL-1573) were cultured and transfected as described [34]. For co-expression studies the plasmids were transfected at a 1∶1 ratio using 5–10 µg DNA per 100-mm culture dish for immunoblotting and immunoprecipitation experiments, and 1–2 µg DNA per 35-mm culture dish for electrophysiology.

### Immunoprecipitation

Transfected HEK293 cells were lysed in extraction buffer (1% Triton X-100, 10 mM Tris-HCl pH7.4, 50 mM NaCl, 1 mM PMSF, 10 µg/ml leupeptin and aprotinin) for 1 h at 4°C. Following centrifugation at 13,000 rpm for 15 min at 4°C in a microfuge, samples were prepared for immunoblotting or the extracts were subject to immunoprecipitation. For anti-Flag-tag immunoprecipitation cell extracts were incubated with M2 antibody (Sigma; 1 µg per 500 µl extract) for 60 min at 4°C. Then, l- or d-glutamate was added to a final concentration of 10 mM, and the incubation was continued for a further 30 min, followed by harvesting the immunocomplexes with GammaBind G Sepharose (GE Healthcare).

### Immunoblotting

Samples were run on 4–12% SDS-polyacrylamide gradient gels (Lonza), transferred to PVDF membrane, and blocked in 3% milk powder/TBS-Tween. Primary antibodies used were: monoclonal anti-Flag M1 (1 µg/ml; Sigma); rabbit anti-stargazin sera (1∶5000; [21]). Horseradish peroxidase -conjugated anti-mouse IgG (1∶3000) or anti-rabbit IgG (1∶3000) (both GE Healthcare) were used as secondary antibodies. ECL signal was detected by exposure to Hyperfilm™ (GE Healthcare) or by the Bio-Rad ChemiDoc XRS system and Quantity One software.

### Whole cell patch clamp electrophysiology

Whole-cell patch-clamp recordings were made as described previously [21], except that EPC 9/2 double patch clamp amplifier and pulse v 8.80 software (HEKA Elektronik, Lambrecht, Germany) were used, and the internal solution contained 140 mM CsCl; 2 mM MgCl_2_; 10 mM EGTA and 10 mM HEPES (pH adjusted to 7.2 with CsOH and osmolality to 305 mOsm with sucrose). Data was analyzed by using Clampfit 10.2 (Molecular Devices, Sunnyvale, CA) and Prism 3.0 softwares (GraphPad, San Diego, CA). Each transfection was done at least twice and 5–8 cells were recorded in each experiment. The data in graphs are presented as mean ± SEM. To determine the rectification index, glutamate-triggered currents were measured at three different holding potentials (−60 mV, 0 mV, +40 mV).

### In vitro RNA synthesis and oocyte electrophysiology

All cRNAs were synthesized *in vitro* by using T7 mMessage mMachine kit from linearized pXOOM templates according to manufacturer's instructions (Ambion, Austin, TX). *Xenopus laevis* oocytes of stages V–VI [37] were injected with cRNAs (total of 0.4–1 ng in 40 nl per oocyte) by using a Nanoject II injector (Drummond, Broomall, PA). For coexpression of stargazin and AMPA receptor subunits, cRNAs were mixed in 1∶1 molar ratio. Oocytes were perfused with 110 mM NaCl, 2 mM KCl, 1 mM MgCl_2_, 10 mM HEPES-NaOH, pH 7.4, and standard two-electrode voltage clamp recordings were performed 1–4 days after cRNA injection at −70 mV holding potential at 20–22°C using TURBO TEC-03X amplifier (npi Electronic GmbH, Tamm, Germany). To analyze inward rectification, currents were recorded at several intermediate voltage clamp values ranging from −70 mV to +70 mV. The electrodes were filled with 3 M KCl and had resistance of 0.8–1.7 MΩ. Agonists were applied for 30–40 s at flow rate of 2 ml/min. Currents evoked by agonist perfusion were filtered at 50 Hz and digitized using CellWorks software (npi electronic).

## Supporting Information

Figure S1 **Concentration–peak glutamate response curves for GluA1i/A2i, GluA1i, and GluA2i receptors.** Concentration-response curves for the l-glutamate triggered peak currents recorded for GluA1i/A2i heteromers, GluA1i homomers and GluA2i homomers. The figure is assembled from curves presented in Fig. 1E and and Fig. 2C for easy comparison.
(PDF)

Figure S2 **GluA4i and stargazin coimmunoprecipitate in the presence and absence of glutamate.** Triton X-100 -extract prepared from HEK293 cells coexpressing GluA4i and stargazin was immunoprecipitated with monoclonal anti-Flag antibody in the continuous presence of l-glutamate (10 mM), d-glutamate (10 mM) or in the absence of glutamate as indicated. Immunocomplexes were resolved in SDS-PAGE and subjected to western blotting by using anti-stargazin antibody and anti-Flag antibody for the detection of stargazin (lower panel) and GluA4i (upper panel), respectively. The experiment was performed four times with similar results.
(PDF)

## References

[1] Kato AS, Gill MB, Yu H, Nisenbaum ES, Bredt DS (2010) TARPs differentially decorate AMPA receptors to specify neuropharmacology. Trends Neurosci 33: 241–248. 20219255 10.1016/j.tins.2010.02.004

[2] Tomita S (2010) Regulation of ionotropic glutamate receptors by their auxiliary subunits. Physiology (Bethesda) 25: 41–49. 20134027 10.1152/physiol.00033.2009 PMC3010179

[3] Jackson AC, Nicoll RA (2011) The expanding social network of ionotropic glutamate receptors: TARPs and other transmembrane auxiliary subunits. Neuron 70: 178–199. 21521608 10.1016/j.neuron.2011.04.007 PMC3119519

[4] Chen L, Chetkovich DM, Petralia RS, Sweeney NT, Kawasaki Y, et al (2000) Stargazin regulates synaptic targeting of AMPA receptors by two distinct mechanisms. Nature 408: 936–943. 11140673 10.1038/35050030

[5] Tomita S, Chen L, Kawasaki Y, Petralia RS, Wenthold RJ, et al (2003) Functional studies and distribution define a family of transmembrane AMPA receptor regulatory proteins. J Cell Biol 161: 805–816. 12771129 10.1083/jcb.200212116 PMC2199354

[6] Kato AS, Zhou W, Milstein AD, Knierman MD, Siuda ER, et al (2007) New transmembrane AMPA receptor regulatory protein isoform, gamma-7, differentially regulates AMPA receptors. J Neurosci 27: 4969–4977. 17475805 10.1523/JNEUROSCI.5561-06.2007 PMC6672084

[7] Kato AS, Siuda ER, Nisenbaum ES, Bredt DS (2008) AMPA receptor subunit-specific regulation by a distinct family of type II TARPs. Neuron 59: 986–996. 18817736 10.1016/j.neuron.2008.07.034

[8] Soto D, Coombs ID, Renzi M, Zonouzi M, Farrant M, et al (2009) Selective regulation of long-form calcium-permeable AMPA receptors by an atypical TARP, gamma-5. Nat Neurosci 12: 277–285. 19234459 10.1038/nn.2266 PMC2735763

[9] Bats C, Groc L, Choquet D (2007) The interaction between Stargazin and PSD-95 regulates AMPA receptor surface trafficking. Neuron 53: 719–734. 17329211 10.1016/j.neuron.2007.01.030

[10] Yamazaki M, Ohno-Shosaku T, Fukaya M, Kano M, Watanabe M, et al (2004) A novel action of stargazin as an enhancer of AMPA receptor activity. Neurosci Res 50: 369–374. 15567474 10.1016/j.neures.2004.10.002

[11] Priel A, Kolleker A, Ayalon G, Gillor M, Osten P, et al (2005) Stargazin reduces desensitization and slows deactivation of the AMPA-type glutamate receptors. J Neurosci 25: 2682–2686. 15758178 10.1523/JNEUROSCI.4834-04.2005 PMC6725153

[12] Tomita S, Adesnik H, Sekiguchi M, Zhang W, Wada K, et al (2005) Stargazin modulates AMPA receptor gating and trafficking by distinct domains. Nature 435: 1052–1058. 15858532 10.1038/nature03624

[13] Turetsky D, Garringer E, Patneau DK (2005) Stargazin modulates native AMPA receptor functional properties by two distinct mechanisms. J Neurosci 25: 7438–7448. 16093395 10.1523/JNEUROSCI.1108-05.2005 PMC6725298

[14] Soto D, Coombs ID, Kelly L, Farrant M, Cull-Candy SG (2007) Stargazin attenuates intracellular polyamine block of calcium-permeable AMPA receptors. Nat Neurosci 10: 1260–1267. 17873873 10.1038/nn1966 PMC2430330

[15] Tomita S, Sekiguchi M, Wada K, Nicoll RA, Bredt DS (2006) Stargazin controls the pharmacology of AMPA receptor potentiators. Proc Natl Acad Sci U S A 103: 10064–10067. 16785437 10.1073/pnas.0603128103 PMC1502506

[16] Tomita S, Byrd RK, Rouach N, Bellone C, Venegas A, et al (2007) AMPA receptors and stargazin-like transmembrane AMPA receptor-regulatory proteins mediate hippocampal kainate neurotoxicity. Proc Natl Acad Sci U S A 104: 18784–18788. 18000041 10.1073/pnas.0708970104 PMC2141854

[17] Menuz K, Stroud RM, Nicoll RA, Hays FA (2007) TARP auxiliary subunits switch AMPA receptor antagonists into partial agonists. Science 318: 815–817. 17975069 10.1126/science.1146317

[18] Cokic B, Stein V (2008) Stargazin modulates AMPA receptor antagonism. Neuropharmacology 54: 1062–1070. 18378265 10.1016/j.neuropharm.2008.02.012

[19] Vandenberghe W, Nicoll RA, Bredt DS (2005) Stargazin is an AMPA receptor auxiliary subunit. Proc Natl Acad Sci U S A 102: 485–490. 15630087 10.1073/pnas.0408269102 PMC544314

[20] Morimoto-Tomita M, Zhang W, Straub C, Cho CH, Kim KS, et al (2009) Autoinactivation of neuronal AMPA receptors via glutamate-regulated TARP interaction. Neuron 61: 101–112. 19146816 10.1016/j.neuron.2008.11.009 PMC2649795

[21] Coleman SK, Moykkynen T, Cai C, von Ossowski L, Kuismanen E, et al (2006) Isoform-specific early trafficking of AMPA receptor flip and flop variants. J Neurosci 26: 11220–11229. 17065461 10.1523/JNEUROSCI.2301-06.2006 PMC6674648

[22] Kott S, Werner M, Korber C, Hollmann M (2007) Electrophysiological properties of AMPA receptors are differentially modulated depending on the associated member of the TARP family. J Neurosci 27: 3780–3789. 17409242 10.1523/JNEUROSCI.4185-06.2007 PMC6672393

[23] Tomita S, Fukata M, Nicoll RA, Bredt DS (2004) Dynamic interaction of stargazin-like TARPs with cycling AMPA receptors at synapses. Science 303: 1508–1511. 15001777 10.1126/science.1090262

[24] Bedoukian MA, Weeks AM, Partin KM (2006) Different domains of the AMPA receptor direct stargazin-mediated trafficking and stargazin-mediated modulation of kinetics. J Biol Chem 281: 23908–23921. 16793768 10.1074/jbc.M600679200

[25] Tomita S, Shenoy A, Fukata Y, Nicoll RA, Bredt DS (2007) Stargazin interacts functionally with the AMPA receptor glutamate-binding module. Neuropharmacology 52: 87–91. 16919685 10.1016/j.neuropharm.2006.07.012

[26] Sager C, Terhag J, Kott S, Hollmann M (2009) C-terminal domains of transmembrane alpha-amino-3-hydroxy-5-methyl-4-isoxazole propionate (AMPA) receptor regulatory proteins not only facilitate trafficking but are major modulators of AMPA receptor function. J Biol Chem 284: 32413–32424. 19773551 10.1074/jbc.M109.039891 PMC2781656

[27] Korber C, Werner M, Hoffmann J, Sager C, Tietze M, et al (2007) Stargazin interaction with alpha-amino-3-hydroxy-5-methyl-4-isoxazole propionate (AMPA) receptors is critically dependent on the amino acid at the narrow constriction of the ion channel. J Biol Chem 282: 18758–18766. 17483093 10.1074/jbc.M611182200

[28] Greger IH, Khatri L, Kong X, Ziff EB (2003) AMPA receptor tetramerization is mediated by Q/R editing. Neuron 40: 763–774. 14622580 10.1016/s0896-6273(03)00668-8

[29] Coleman SK, Möykkynen T, Hinkkuri S, Vaahtera L, Korpi ER, et al (2010) Ligand binding domain determines endoplasmic reticulum exit of AMPA receptors. J Biol Chem 285: 36032–36039. 20837486 10.1074/jbc.M110.156943 PMC2975225

[30] Lu W, Shi Y, Jackson AC, Bjorgan K, During MJ, et al (2009) Subunit composition of synaptic AMPA receptors revealed by a single-cell genetic approach. Neuron 62: 254–268. 19409270 10.1016/j.neuron.2009.02.027 PMC3632349

[31] Nakagawa T, Cheng Y, Ramm E, Sheng M, Walz T (2005) Structure and different conformational states of native AMPA receptor complexes. Nature 433: 545–549. 15690046 10.1038/nature03328

[32] Yan D, Tomita S (2012) Defined criteria for auxiliary subunits of glutamate receptors. J Physiol 590: 21–31. 21946847 10.1113/jphysiol.2011.213868 PMC3300042

[33] Schwenk J, Harmel N, Zolles G, Bildl W, Kulik A, et al (2009) Functional proteomics identify cornichon proteins as auxiliary subunits of AMPA receptors. Science 323: 1313–1319. 19265014 10.1126/science.1167852

[34] Pasternack A, Coleman SK, Jouppila A, Mottershead DG, Lindfors M, et al (2002) Alpha-amino-3-hydroxy-5-methyl-4-isoxazolepropionic acid (AMPA) receptor channels lacking the N-terminal domain. J Biol Chem 277: 49662–49667. 12393905 10.1074/jbc.M208349200

[35] Ho SN, Hunt HD, Horton RM, Pullen JK, Pease LR (1989) Site-directed mutagenesis by overlap extension using the polymerase chain reaction. Gene 77: 51–59. 2744487 10.1016/0378-1119(89)90358-2

[36] Jespersen T, Grunnet M, Angelo K, Klaerke DA, Olesen SP (2002) Dual-function vector for protein expression in both mammalian cells and Xenopus laevis oocytes. Biotechniques 32: 536–538, 540. 11911656 10.2144/02323st05

[37] Liu XS, Liu XJ (2006) Oocyte isolation and enucleation. Methods Mol Biol 322: 31–41. 16739714 10.1007/978-1-59745-000-3_3

