# Supplementary figures and images for: Autoinactivation of the Stargazin–AMPA Receptor Complex: Subunit-Dependency and Independence from Physical Dissociation
Source: PLoS One. 2012 Nov 14;7(11):e49282. doi: 10.1371/journal.pone.0049282 (PMC3498123; doi:10.1371/journal.pone.0049282)

Supporting information - Figure S1

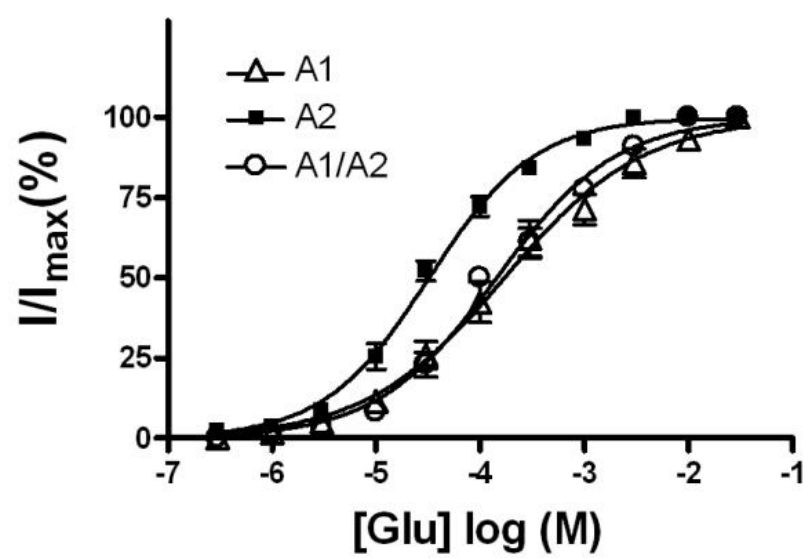

Supplement: Figure S1 — Concentration–peak glutamate response curves for GluA1i/A2i, GluA1i, and GluA2i receptors. Concentration-response curves for the l-glutamate triggered peak currents recorded for GluA1i/A2i heteromers, GluA1i homomers and GluA2i homomers. The figure is assembled from curves presented in Fig. 1E and and Fig. 2C for easy comparison. (PDF) [file pone.0049282.s001.pdf]

Supporting information - Figure S2

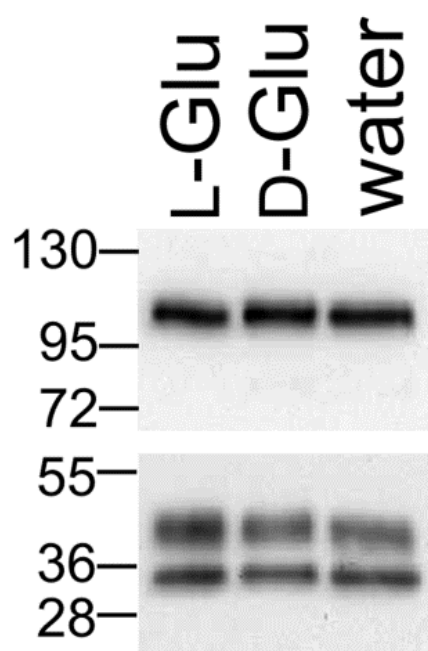

Supplement: Figure S2 — GluA4i and stargazin coimmunoprecipitate in the presence and absence of glutamate. Triton X-100 -extract prepared from HEK293 cells coexpressing GluA4i and stargazin was immunoprecipitated with monoclonal anti-Flag antibody in the continuous presence of l-glutamate (10 mM), d-glutamate (10 mM) or in the absence of glutamate as indicated. Immunocomplexes were resolved in SDS-PAGE and subjected to western blotting by using anti-stargazin antibody and anti-Flag antibody for the detection of stargazin (lower panel) and GluA4i (upper panel), respectively. The experiment was performed four times with similar results. (PDF) [file pone.0049282.s002.pdf]
